# *Vibrio frigidus* sp. nov., Isolated from the Gills of Atlantic Salmon (Salmo salar)

**DOI:** 10.1007/s00284-026-05041-7

**Published:** 2026-07-10

**Authors:** Rannveig Hrólfsdóttir, Viggó Þór Marteinsson, Gunnsteinn Haraldsson, Snædís Björnsdóttir, Eva Benediktsdóttir

**Affiliations:** 1 Institute of Life and Environmental Sciences, University of Iceland, Reykjavík, Iceland; 2 Food Safety and Environment, Matís ohf, Reykjavík, Iceland; 3 Faculty of Food Science and Nutrition, University of Iceland, Reykjavík, Iceland; 4 Faculty of Medicine, University of Iceland, Reykjavík, Iceland; 5 Department of Clinical Microbiology, The National University Hospital of Iceland, Reykjavík, Iceland

## Abstract

A novel strain, designated F74^T^, was isolated from the gills of a healthy Atlantic salmon, *Salmo salar* L., at a land-based aquaculture farm located on the Reykjanes peninsula in Iceland. The bacteria were facultative anaerobic, Gram-stain-negative curved rods that were motile and required NaCl for growth. They were sensitive to the vibriostatic agent O/129 and showed growth at 0 °C. The predominant fatty acids were C_16:1_ ω7c and/or C_15_ iso 2-OH (summed feature 3) (53.5%) and 16:0 (16.7%). The digital genomic DNA G + C content was 41.6%. Comparative analysis of the 16S rRNA gene revealed that the type strain most closely related to F74^T^ was *Vibrio algarum* KJ40-1^T^ with a 16S rRNA gene sequence similarity of 98.4%. Other type strains presented similarity of less than 96%. Phylogenetic analysis based on 16S rRNA genes revealed that F74^T^ represented a member of the genus *Vibrio* and exhibited the closest relatedness to *V. algarum* KJ40-1^T^, which was further confirmed by a phylogenetic analysis based on eight housekeeping genes. *In silico* DNA–DNA hybridization and ANI comparison of closely related species were far below the corresponding thresholds for discriminating bacterial species. Based on phenotypic, chemotaxonomic and phylogenetic evidence, F74^T^ is considered to represent a novel species of the genus *Vibrio*, for which the name *Vibrio frigidus* sp. nov. is proposed. The type strain is F74^T^ (= DSM 23163^T^ = NCIMB 14608^T^).

## Introduction

The genus *Vibrio*, which currently consists of 156 species with validly published name, (https://lpsn.dsmz.de/family/vibrionaceae, accessed Jan. 26th, 2026) [1] is a member of the family *Vibrionaceae*, order *Vibrionales*, phylum *Pseudomonadota*. The members of the genus *Vibrio* are Gram-stain-negative straight or curved rods that are facultatively anaerobic and usually oxidase positive, motile and halophilic. It is considered one of the most diverse and well-studied groups of bacteria, and most members inhabit marine and estuarine environments [2]. Many *Vibrio* species are well known as important pathogens in humans, where they are often associated with the consumption of seafood or sea baths [3]. Many *Vibrio* species are pathogenic to marine organisms in aquaculture worldwide, but vibrios are also known to constitute a normal part of their microbiome [4].

The majority of *Vibrio* species hitherto described are mesophilic. However, in places where the sea temperature is less than 15 °C most of the year and less than 5 °C during the winter months, cold-adapted bacteria flourish, and in such environments the psychrotrophs are important pathogens in fish farming [5, 6]. *Vibrio* species that can grow at low temperatures have also been isolated from places such as food production facilities, where low temperatures are desirable [7–9].

In a study performed on cold-adapted bacteria of the family *Vibrionaceae* associated with fish and seawater, a bacterial strain designated F74^T^ attracted our attention because of its fermentative ability compared with the other psychrotrophic bacteria isolated. The strain F74^T^ was isolated from the gills of Atlantic salmon, on selective media after enrichment. The gills are a gateway into the fish body and are continuously exposed to the microorganisms present in the surrounding water, which entails uncertainty about if the strain is presenting a minor part of the resident microbial community on the gills of Atlantic salmon or a transient flora originating in the water.

In this study we characterized this strain using genomic, phylogenetic, chemotaxonomic and physiological analysis to determine its taxonomic properties.

## Materials and Methods

### Strain Isolation

The strain designated F74^T^ was isolated on May 7th 1993, from the gills of a healthy salmon in a land-based flow-through salmon farm in southwestern Iceland (63° 48’ 59’’ N 22° 31’ 56’’ W). Brackish water with stable temperature and salinity was pumped from an eight-meter-deep borehole in a lava bedrock, aerated and then pumped into the tanks. Tank water was maintained at 6.5 °C and a salinity of 29‰, monitored regularly using a mercury thermometer and a hydrometer. Fish were captured by net and immediately killed by a sharp blow to the head, placed in a sterile plastic bag and transported to the laboratory for sampling. The operculum and the uppermost gill arch were cut off and discarded, and the next three gill arches were cut off and irrigated with 50 ml of sterile 2% (w/v) NaCl. The filaments were cut from the arches, weighed and homogenized in a tenfold volume of 2% (w/v) NaCl, using a Stomacher^®^ Lab-Blender 400. One ml of the homogenate was inoculated in an enrichment medium composed of electrolyte supplemented with alkaline peptone water (10 g peptone, 15 g NaCl, 7 g MgSO_4_ · 7 H_2_O, 0.75 g KCl, 0.01 g CaCl_2_ and 1000 ml distilled water; pH 8.6) and incubated for 48 h at 15 °C. A loopful was streaked on thiosulfate-citrate-bile salts-sucrose agar (TCBS, Oxoid, Basingstoke, UK) and cultivated for 72 h at 15 °C; subsequently, a yellow colony, 1 mm in diameter, was picked and cultivated on Marine Broth 2216^®^ (Difco, Detroit, Michigan, USA) with 1.2% (w/v) agar (Agar No 1, Oxoid, Basingstoke, UK) (MA).

### Physiological and Biochemical Characteristics

Biochemical and physiological tests were performed as described previously, using type strains of *Vibrio*, *Aliivibrio* and *Moritella* as reference [10]. The tests included the following: sensitivity for O129, oxidase, catalase, oxidative and fermentative degradation of glucose, gas production, luminescence, growth at 0, 0.5, 1, 2, 3, 4, 5, 6, 7 and 8% (w/v) NaCl; growth at 4, 10, 15, 21, 24, 26, 30 and 37 °C; production of arginine dehydrogenase, lysine and ornithine decarboxylases; nitrate reduction; indole production; production of acetoin (Voges–Proskauer test); oxidative production of acid from arabinose; cellobiose, glycerol, inositol, lactose, maltose, mannose, mannitol, melibiose, N-acetyl-glucosamine, rhamnose, ribose, sucrose, salicin, sorbitol and trehalose; and degradation of starch, gelatin, DNA, Tween 20, Tween 80 and chitin. For estimation of the optimum temperature, growth at 10, 14, 17 and 20 °C was examined in Tryptic Soy Broth (Difco, Detroit, Michigan, USA) supplemented with 2% NaCl and turbidity was read 12 times at eight to 15 h interval up to 161 h. The growth at 0 °C was estimated with the culture flasks incubated in icewater, and turbidity was read at two- or three- days intervals up to 32 days. The turbidity was measured in 96–well microtiter plates, 300 µl of suspension were put into each well and the optical density was read at 590 nm in EMax^®^ absorbance microplate reader (Molecular devices).

### Scanning Electron Microscopy

The cell morphology was examined by scanning electron microscopy (SEM) using Leo Supra 25 field emission scanning electron microscope (FE-SEM). Strain F74^T^ was cultivated in Marine Broth (MB) at 10 °C for four days. The cells were fixed in 2% glutaraldehyde, dehydrated through an ethanol series, critical point dried and coated with gold using an Edwards Sputter Coater S150B.

### Chemotaxonomic Analysis

Quantitative analysis of the cellular fatty acid composition was performed by DSMZ, Germany, after cultivation of the cells on MA at 4 °C for 4 days. A loop of cell mass was harvested, and the fatty acids were saponified, methylated and extracted according to the protocol of the Sherlock Microbial Identification System (MIDI), analysed by gas chromatography and identified using the Microbial Identification software package [11].

### 16S rRNA and Genome Sequencing

For sequencing of the 16S rRNA gene, genomic DNA was isolated with a DNA extraction kit (Dynabeads, Dynal ASA, Oslo, Norway). The 16S rRNA gene was amplified with the primers F9 and R1544 as previously described [12]. Nucleotide sequences were determined using an Applied Biosystems 3730 DNA analyser and a BigDye terminator cycle sequencing kit. The primers F9 and R1544 were used for sequencing, as were the primers F338, R515, R805 and R1195 [12].

For whole-genome sequencing, a loopful of bacteria was suspended in sterile PBS, and DNA was extracted using the MagNAPure 96 (Roche). Sequencing, assembly and annotation were performed by MicrobesNG, Birmingham, UK, (microbesng.com) using Illumina NovaSeq and SPAdes3.7 [13–14]. The contamination and completeness of the genome were assessed using CheckM2 (ver. 1.1.0) [15].

### Phylogenetic and Genomic Analysis

The determined 1,439 bp 16S rRNA gene sequence was subjected to comparison in GenBank using BLAST limited to type material of *Vibrio* species and BLAST 2 at the National Library of Medicine (www.ncbi.nlm.nih.gov). For further analysis, 38 reference strains were selected based on the outcome of the BLAST comparisons and also the results obtained by Jiang et al. [16] and Butt et al. [17]. The 16S rRNA gene sequences of the strains were obtained from GenBank (www.ncbi.nlm.nih.gov) and aligned with the corresponding sequence of F74^T^. Fragments that corresponded to *E. coli* K-12 sequences at positions 194 to 1,403 [18] were used for the reconstruction of a phylogenetic tree. The CLUSTALW program implemented in MEGA 11 was used for the alignments. Phylogenetic trees were reconstructed using MEGA 11 with both the maximum likelihood and the neighbour-joining method with bootstrap analyses based on 1000 replicates [19].

The 16S rRNA trees were used to select type strains for the calculations of *in silico* DNA-DNA hybridization (dDDH) values and the orthologous average nucleotide identity (ANI). The dDDH values were calculated using the Type Strain Genome Server (TYGS web server, https://tygs.dsmz.de) [1] and the ANI values were calculated using the Orthologous Average Nucleotide Identity Tool software [20] available on the EzBioCloud server.

The phylogenetic tree was also used to select 16 type strains for multilocus sequence analysis (MLSA). Eight housekeeping genes, *ftsZ*,* gapA*,* gyrB*,* mreB*,* pyrH*,* recA*,* rpoA* and *topA* [16], were retrieved from annotated genomes from GenBank (www.ncbi.nlm.nih.gov/datasets) and from the sequence of the strain F74^T^, based on the corresponding gene or protein annotations. The flanking ends were trimmed, and the fragments were concatenated according to Jiang et al. [16] (*Vibrio cholerae* 14035^T^ ungapped gene numbering), producing a multilocus sequence alignment of 9,205 positions. The same algorithms were used for the alignments and the reconstruction of phylogenetic trees as for the trees based on the 16S genes sequences.

At a late stage of the study, a genome-wide search for cold-adaptation genes in F74^T^ was conducted and thirty putative genes were subjected to BLAST analysis (www.ncbi.nlm.nih.gov) against *Gammaproteobacteria* in GenBank. This analysis identified two unclassified *Vibrio* strains, *Vibrio* sp. VB16 (NZ_CP087590.1) and *Vibrio* sp. DW001 (NZ_CP091975.1), which were subsequently included in the calculation of dDDH and ANI values using the same methods as applied for the type strains.

## Results and Discussion

### Morphological, Physiological and Biochemical Characteristics

**Fig. 1:**
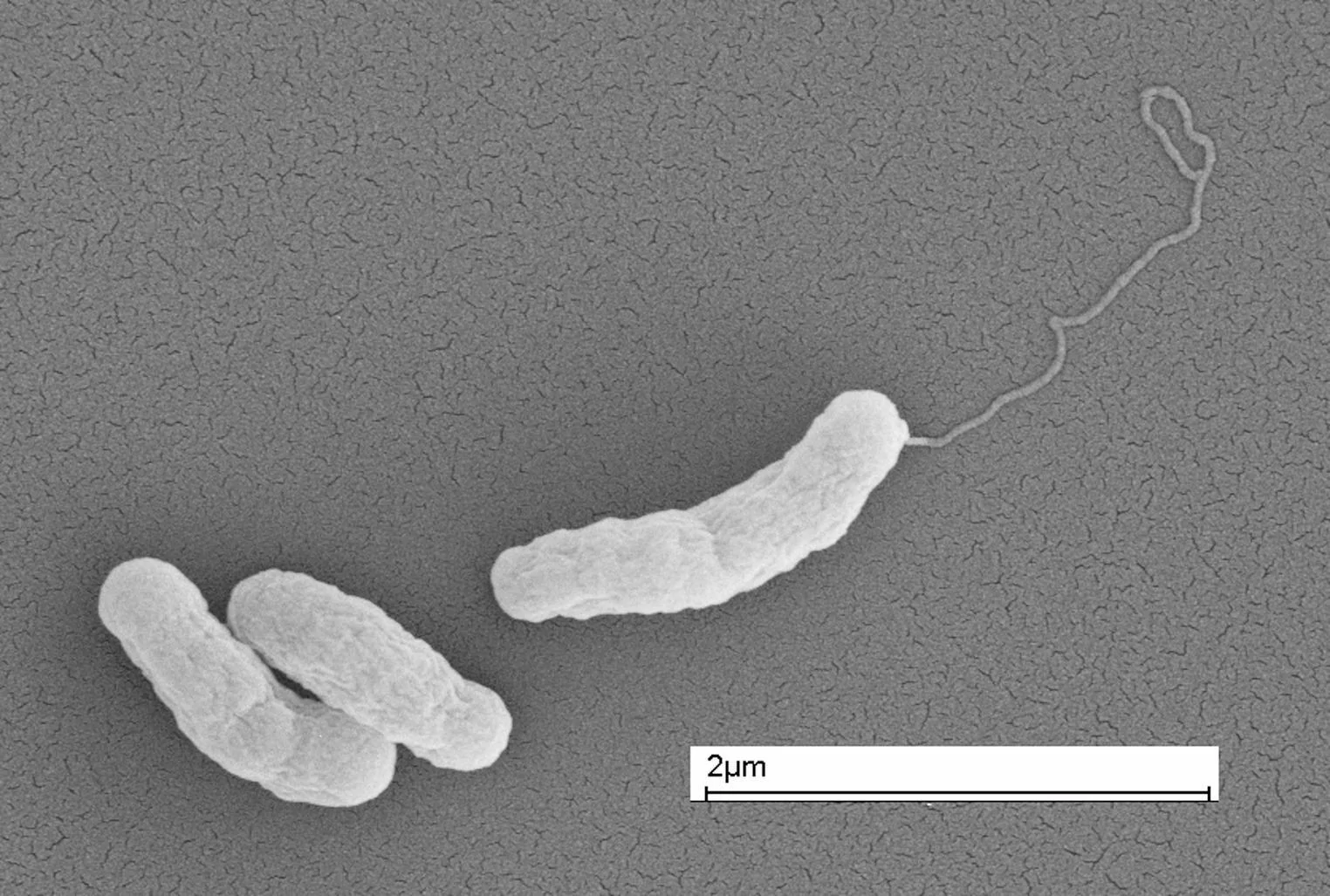
Scanning electron micrograph of F74^T^ showing cell shape and the polar flagellum

Scanning electron microscopy revealed that the F74^T^ cells were slightly curved rods with one polar flagellum (Fig. 1). They were Gram-stain-negative, of approximately 1–2 μm length and 0.2–0.4 μm width. The MA colonies were approximately 2 mm in diameter after 48 h of incubation at 15 °C, and on the TCBS agar colonies were tiny, rounded and yellow. Growth occurred at 0 to 24 °C and in media supplemented with 1‒5% (w/v) NaCl. Optimum temperature for growth was around 10 °C. The highest maximum density, 0.545, was obtained at 0 °C after 16 days of cultivation. The highest density obtained at 20 °C was 0.147, after 84 h of cultivation. Other phenotypic characteristics are given in the species description.

### Chemotaxonomic Features

The fatty acid content of strain F74^T^ was as follows: C_16:1_ ω7c and/or C_15_ iso 2-OH (summed feature 3), 53.5%; C_16:0_, 16.7%; C_18:1_ω7c, 12.0%; C_12:0_, 4.2%; C_12:0_ 3-OH, 3.6%; C_14:0_, 1.8%; C_14:0_ 3-OH and/or iso-C_16:1_ (summed feature 2), 1.7%. The composition of the fatty acids is similar to the fatty acid profiles of related species of the genus *Vibrio* reported by Butt et al. [17], but exact comparisons are difficult between species that differ in cultivation temperatures. However, it is noteworthy that the proportion of C_16:1_ ω7c and/or iso-C_15:0_ 2-OH (summed feature 3) is considerably larger in F74^T^ (53.5%) than in the related *Vibrio* species (30.3‒41.9%) [17].

### Genomic Features and Phylogeny

Comparative analysis of the 16S rRNA gene using BLAST revealed that the strain most closely related to F74^T^ was *Vibrio algarum* KJ40-1^T^, with a 16S rRNA gene sequence similarity of 98.4%. All other strains showed similarity values of less than 96%, i.e. *Vibrio hannami* 168GH5-2-16^T^ and *Vibrio ulleungensis* 188UL20-2^T^, which showed 95.5% and 95.3% similarity, respectively.

**Fig. 2:**
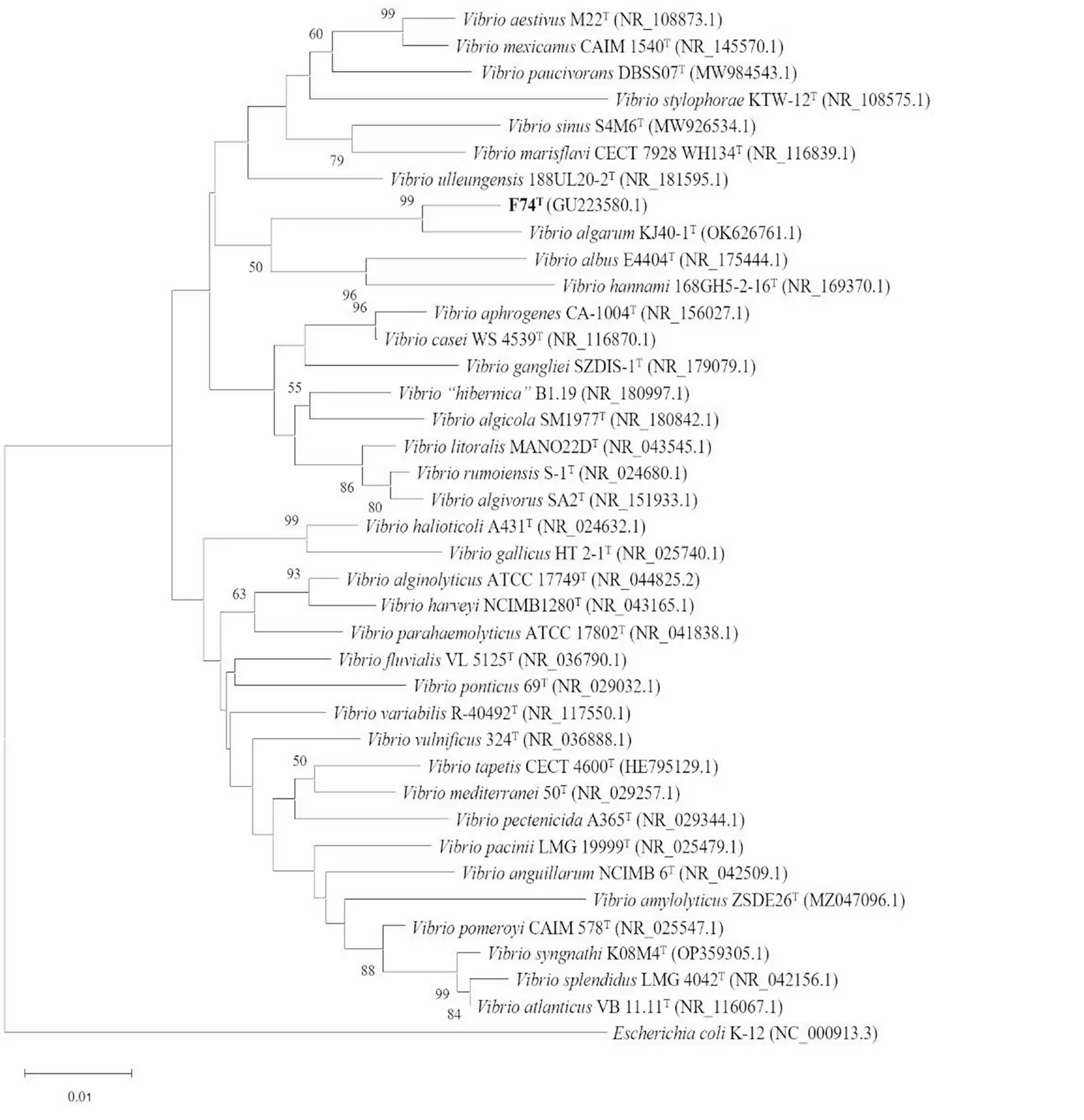
Neighbour-joining tree based on 16S rRNA gene sequences showing the phylogenetic relationship of strain F74^T^ and related species of the genus *Vibrio.* Bootstrap values greater than or equal to 50%, based on 1000 replicates, are indicated at the branching points. *E. coli* K-12 MG 1655 (NC_ 000913.3) was used as an outgroup

Phylogenetic analysis using the neighbour-joining method based on the 16S rRNA sequences of 38 *Vibrio* strains revealed that 19 strains, consisting of F74^T^ and related strains, formed a phylogenetic lineage away from 19 *Vibrio* strains that had been selected as representatives of different clades of the genus *Vibrio* [16] (Fig. 2). A phylogenetic tree based on the same sequences computed with the maximum likelihood method showed comparable results.

The draft genome size was 5,025,028 bp, the total number of contigs was 136, the number of coding sequences (CDSs) was 4,513, the N_50_ was 358,377 and the number of RNA genes was 105, thereof 79 tRNA genes. The digital genomic DNA G + C content was 41.6%. Assessing the completeness and contamination of the genome using CheckM2 resulted in 100% completeness and 0.4% contamination [15].

The highest ANI values were found between F74^T^ and *V. algarum* KJ40-1^T^, *V. hannami* KACC 19,277 ^T^ and *Vibrio albus* E4404 ^T^, with values of 79.7, 71.9 and 71.8%, respectively.

The highest correlation of dDDH values was detected between strain F74^T^ and *Vibrio algicola* SM1977 ^T^, *Vibrio aphrogenes* CA-1004^T^, *Vibrio rumoiensis* FERM P-14,531^T^ and *V. algarum* KJ40-1^T^, with values of 24.0, 23.9, 22.9 and 22.7%, respectively. These values are all below the proposed cut-off values for species delineation, which are 95‒96% for ANI and 70% for dDDH [21].

**Fig. 3:**
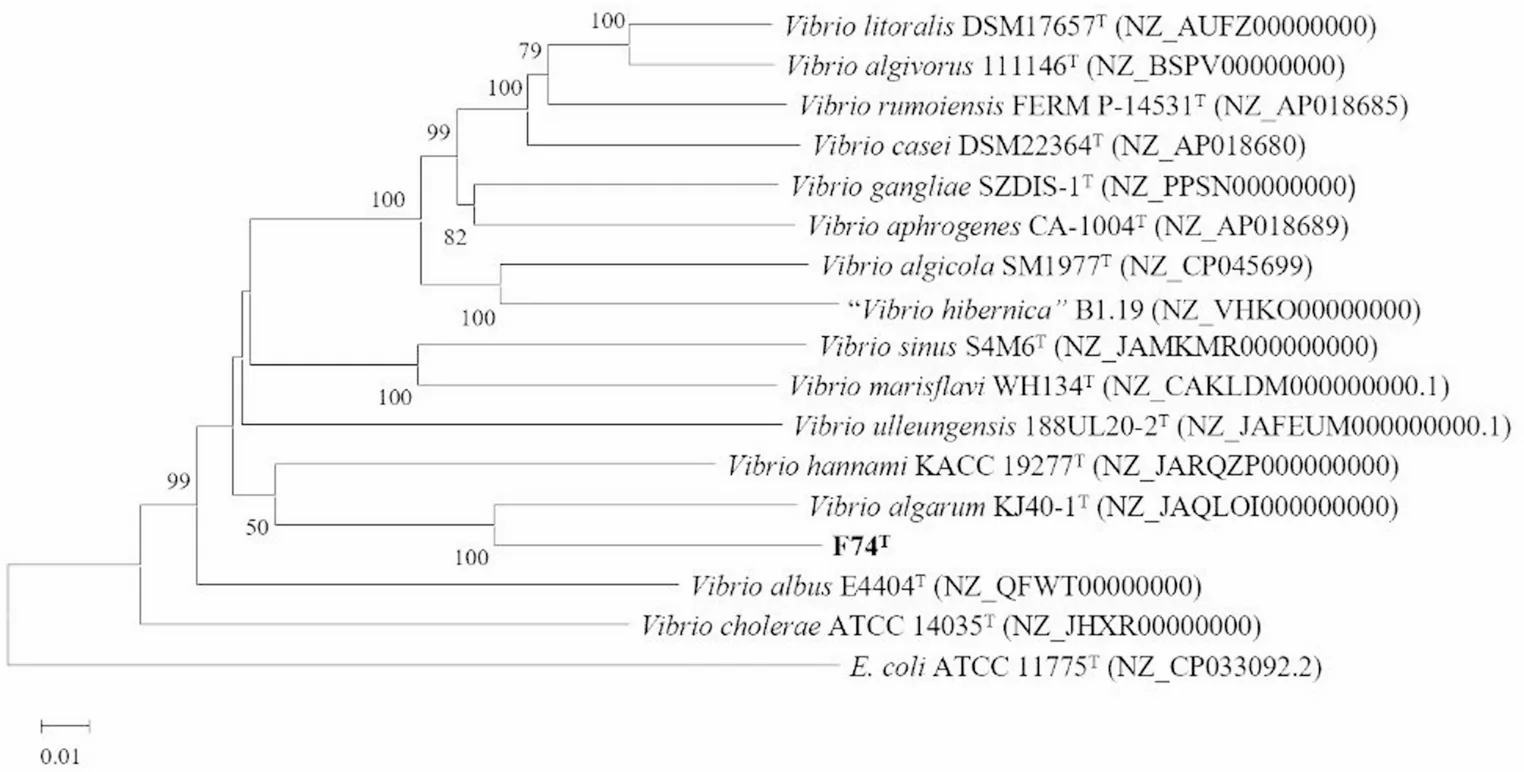
Neighbour-joining tree based on sequences of eight housekeeping genes, *ftsZ*,* gapA*,* gyrB*,* mreB*,* pyrH*,* recA*,* rpoA* and *topA*, showing the phylogenetic relationships of strain F74^T^ and related species of the genus *Vibrio*. Bootstrap values greater than or equal to 50%, based on 1000 replicates, are indicated at the branching points. *E. coli* ATCC 11,775^T^ (CP033092.2) was used as an outgroup

To further verify the taxonomic position of F74^T^, multilocus sequence analysis (MLSA) was performed, using eight housekeeping genes, *ftsZ*,* gapA*,* gyrB*,* mreB*,* pyrH*,* recA*,* rpoA* and *topA*, for F74^T^ and the most related *Vibrio* strains according to the above results. Figure 3 shows the results when the neighbour‒joining method was used and reveals a phylogenetic lineage of F74^T^ and *V. algarum* KJ40-1^T^. A phylogenetic tree that was reconstructed using the maximum likelihood method showed similar results.

Psychrotrophic bacteria must possess features that enable growth at low temperatures. Since F74^T^ is capable of growing at 0 °C, a genome-wide analysis was conducted to examine the presence of putative cold-adaptation genes. Genome annotation revealed 37 such genes, of which 30 were subjected to BLAST comparison within the *Gammaproteobacteria*. All comparisons except one yielded 88.5–100% identity with homologous genes in two sequenced genomes belonging to strains lacking species designation, *Vibrio* sp. VB16 (NZ_CP087590.1) and *Vibrio* sp. DW001 (NZ_CP091975.1). In eleven cases, genes from these two strains were the only or sole matches retrieved, with one or two additional strains appearing in three of those cases. These potential cold-adaptation genes included seven encoding universal stress proteins, two encoding proteins involved in osmotic stress response, one encoding a desaturase and one encoding a catalase. Of the remaining 19 genes subjected to BLAST analysis, more than 70% sequence identity was found with genes from mesophilic marine species, most often *Vibrio* spp., while in three cases, 80–92% similarity was found primarily with genes of marine bacteria belonging to other genera, often psychrotrophic.

The results indicate that F74^T^ is well adapted to life in cold environments and highlight gene products that may be restricted to psychrotropic *Vibrio* species. Notably, the two *Vibrio* strains sharing the remarkably high sequence similarity with the F74^T^ were both isolated from cold environments: DW001 isolated from Antarctic sea ice and VB16 from intertidal sediment during winter at Virginia Beach, USA [22]. This raises the question of whether these strains belong to the same species as F74^T^. ANI and dDDH values between F74^T^ and DW001 were 94.4% and 58.1%, respectively, while the corresponding values between F74^T^ and VB16 were 94.1% and 56.1%. Although both ANI and dDDH values fall below the thresholds for species delineation, they approach these limits closely. Furthermore, a BLAST search of the 16S rRNA gene, when not restricted to type strains, revealed greater than 99% sequence similarity between F74^T^ and both strains.

Taken together, the findings presented here support the conclusion that F74^T^ represents a novel species within the genus *Vibrio.* The analysis, that was limited to described species, revealed a robust lineage with *Vibrio algarum*, which is supported by the similarity of the G + C content and the genome size of F74^T^ and *V. algarum* KJ40-1^T^ (Table 1).

**Table 1:** Differential features of strain F74^T^ and related species of the genus *Vibrio*

Characteristic	1	2	3	4	5	6	7	8	9
Origin	Salmon	Algae	Seawater	Meat brine	Fish proc. plant	Sediment	Mussel	Seawater	Soft cheese
Genome length (MB)	5.0	4.9	5.4^1)^	3.4	4.2^1)^	4.3	4.4	4.8^1)^	4.3
G + C%	41.6	40.8	43.8^2)^	40.6	43.2	46.1	45.4	42.5	41.8
Motility	+	−	+	NR	−	+	+	+	+
Catalase	+	+	+	−	+	+	+	+	+
Temperature range (°C)	0‒24	10‒30	20‒42	4‒23	2‒34	15‒37	18‒37	16‒37	2‒30
NaCl range (%)	1‒5	1‒8	1‒8	0‒10	3‒6	1‒6	1‒4	1‒6	2‒10
Acetoin production	+	+	+	NR	−	−	−	−	NR
Arginine dihydrolase	+	−	+	−	−	−	−	+	NR

Strains: 1 F74^T^, 2 *Vibrio algarum* KJ40-1^T^, 3 *Vibrio hannami* KACC 19,277 ^T^, 4 “*Vibrio hibernica*” B1.19, 5 *Vibrio rumoiensis* S-1^T^, 6 *Vibrio albus* E4404^T^, 7 *Vibrio ulleungensis* 188UL20-2^T^, 8 *Vibrio marisflavi *WH134^T^, 9 *Vibrio casei* WS4539^T^

Data for F74^T^ is from the present study. Other data presented are taken from the following references, with three exceptions (see footnotes): Data for *Vibrio algarum* KJ40-1^T^ [17]; *Vibrio hannami *KACC 19,277 ^T^ [23]; “*Vibrio hibernica*” B1.19 [8]; *Vibrio rumoiensis* S-1 ^T^ [7]; *Vibrio albus* E4404^T^ [24]; *Vibrio ulleungensis* 188UL20-2^T^ [25]; *Vibrio marisflavi* WH134^T^ [26];* Vibrio casei* WS4539^T^ [9].

^1^Data obtained from GenBank (https://www.ncbi.nlm.nih.gov); ^2)^ Data obtained from Butt et al. [17]; NR: Not reported; + Positive result; − Negative result.

The genotypic and phenotypic properties of F74^T^, *V. algarum* KJ40-1^T^ and related species are presented in Table 1. Few of the most closely related species can grow at 4 °C, but all except “*Vibrio hibernica*” are able to grow at 30 °C or even 40 °C or higher. F74^T^ differs from “*V. hibernica*” in the production of catalase and salt tolerance, as “*V. hibernica*” is catalase negative and able to grow in Tryptic Soy Broth with no added NaCl and up to 10% (w/v) NaCl, whereas F74^T^ is catalase positive and grows from 1 to 5% (w/v) NaCl.

Within the genus *Vibrio*, the closest relative of F74^T^ is *V. algarum* [17]. They can be differentiated by several traits: F74^T^ has a polar flagellum and is motile, but *V. algarum* KJ40-1^T^ is nonmotile. F74^T^ grows at temperatures up to 24 °C and NaCl concentrations of up to 5% (w/v), whereas *V. algarum* KJ40-1^T^ can grow at 30 °C and NaCl concentrations of 8% (w/v). F74^T^ is positive for arginine dihydrolase, whereas *V. algarum* KJ40-1^T^ is negative.

## Taxonomic Conclusion

On the basis of phylogenetic, genotypic, phenotypic and chemotaxonomic evidence, strain F74^T^ is concluded to represent a novel species of the genus *Vibrio*, for which the name *Vibrio frigidus* sp. nov. is proposed.

### Description of *Vibrio frigidus* sp. nov

*Vibrio frigidus* [fri’gi.dus. L. masc. adj. *frigidus*, cold, referring to growth in a cold environment].

Cells are slightly curved Gram-stain-negative rods, approximately 1–2 μm long and 0.2–0.4 μm wide and motile by a polar flagellum. Non-luminescent. Colonies are approximately 2 mm in diameter on marine agar after 48 h of incubation at 15 °C, translucent, convex, nonswarming and rounded with entire margins, but tiny, rounded and yellow on thiosulfate-citrate-bile salts-sucrose agar. Growth occurs at 0–24 °C (optimum, 10 °C) and in the presence of 1–5% (w/v) NaCl.

Produces catalase and cytochrome c oxidase. Nitrate is reduced to nitrite. Indole is produced from tryptophan. Does not produce acetoin. Produces arginine dihydrolase but not ornithine- and lysine decarboxylases. Acid is produced from the following compounds: arabinose, cellobiose, glucose, glycerol, lactose, maltose, mannitol, mannose, melibiose, N-acetylglucosamine, ribose, sucrose, sorbitol and trehalose. Does not produce acid from rhamnose and salicin. Produces gelatinase and DNase but not amylase and chitinase. Gelatine, DNA, Tween 20 and Tween 80 are hydrolyzed but starch and chitin are not. The major fatty acids are summed feature 3 (C_16:1_ ω7c and/or C_15_ iso 2-OH), C_16:0_, and C_18:1_ω7c.

The type strain F74^T^ (= DSM 23163^T^ = NCIMB 14608^T^) was isolated from the gills of a healthy salmon at a land-based farm in the Reykjanes Peninsula in southwestern Iceland. The type strain genome size is 5.03 Mb and DNA G + C is 41.6% (calculated from the whole genome sequence). The 16S rRNA gene sequence and the genomic sequence of strain F74^T^ have been deposited under the GenBank accession numbers GU223580.1 and JBCDMC000000000.1, respectively.

## Data Availability

The type strain F74^T^ is publicly available in Leibniz Institute DSMZ-German Collection of Microorganisms and Cell Cultures GmbH under the accession DSM 23163^T^, and the UK National Collection of Industrial, Food and Marine Bacteria under the accession NCIMB 14608^T^. The 16S rRNA gene sequence and the genomic sequence of strain F74^T^ have been deposited under the GenBank accession numbers GU223580.1 and JBCDMC000000000.1, respectively.
